# Thermally stable amorphous tantalum yttrium oxide with low IR absorption for magnetophotonic devices

**DOI:** 10.1038/s41598-017-14184-4

**Published:** 2017-10-23

**Authors:** Takuya Yoshimoto, Taichi Goto, Hiroyuki Takagi, Yuchi Nakamura, Hironaga Uchida, Caroline A. Ross, Mitsuteru Inoue

**Affiliations:** 1 Department of Electrical and Electronic Information Engineering, Toyohashi University of Technology, 1-1 Hibari-Ga-Oka, Tempaku, Toyohashi, Aichi 441-8580 Japan; 2 JST, PRESTO, 4-1-8 Honcho, Kawaguchi, Saitama, 332-0012 Japan; 3 Department of Materials Science and Engineering, Massachusetts Institute of Technology, 77 Massachusetts Avenue, Cambridge, Massachusetts, 02139 USA

## Abstract

Thin film oxide materials often require thermal treatment at high temperature during their preparation, which can limit them from being integrated in a range of microelectronic or optical devices and applications. For instance, it has been a challenge to retain the optical properties of Bragg mirrors in optical systems at temperatures above 700 °C because of changes in the crystalline structure of the high–refractive-index component. In this study, a ~100 nm–thick amorphous film of tantalum oxide and yttrium oxide with an yttrium-to-tantalum atomic fraction of 14% was prepared by magnetron sputtering. The film demonstrated high resistance to annealing above 850 °C without degradation of its optical properties. The electronic and crystalline structures, stoichiometry, optical properties, and integration with magnetooptical materials are discussed. The film was incorporated into Bragg mirrors used with iron garnet microcavities, and it contributed to an order-of-magnitude enhancement of the magnetooptical figure of merit at near-infrared wavelengths.

## Introduction

High temperature processing is essential to the preparation of high-quality functional oxides that exhibit magnetism^1,2^, ferroelectricity^3^, room-temperature multiferroicity^4,5^, and low optical absorption^6^. These properties are used in a range of magnetooptical^7^, spintronic^8^, magnonic^9,10^, ionic^11–13^, and multiferroic devices^4^. To integrate materials that require high temperature processing into heterostructures and devices, the thermal stability of other materials in the device is a basic requirement. In particular, integrating functional oxides into optical components—e.g., Bragg mirrors (BMs), one of the best-known optical devices, which are made from alternating layers with high and low refractive indices (*n*)—is challenging because of the increase in optical losses in the high-*n* material caused by high temperature annealing.

Tantalum oxide (Ta_2_O_5_) is widely used as a high-*n* material in BMs because of its high transmissivity and high heat resistance^14,15^. Compared with other high-*n* materials, such as titanium oxide (TiO_2_)^16^ and hafnium oxide (HfO_2_)^17,18^, Ta_2_O_5_ has crystallisation and glass-transition temperatures that are approximately 300 °C higher^19^. Despite this, Ta_2_O_5_ lacks the necessary thermal stability to withstand the annealing required for integration of magnetooptical oxides, such as iron garnets, which may require an 800 °C oxygen annealling in order to fully crystallise and exhibit high Faraday rotation. For example, degradation of the quality of a high-*n* material decreased the performance of a microcavity, comprising a magnetooptical layer sandwiched between two BMs^19–21^.

We previously demonstrated a microcavity in which a cerium-substituted yttrium iron garnet film (CeYIG, Ce_x_Y_3–x_Fe_5_O_12_, with x ~1.0) was used as the magnetooptical material and Ta_2_O_5_ and SiO_2_ were used in the BMs^19^. The Faraday rotation of the microcavity was 30 times that of the CeYIG film at the localization wavelength; however, the Faraday rotation and transmissivity were lower than the theoretical values calculated by the matrix approach^22^. This degradation was attributed to the crystallisation of the Ta_2_O_5_ used in the BM during the 800 °C anneal that was performed to crystallise the CeYIG. Hence, to improve the Faraday rotation and transmissivity of the microcavity, the crystallisation of Ta_2_O_5_ must be suppressed.

Crystallisation may be suppressed by adding other elements whose ionic radii are larger than those of elements in the base material^23–26^. Toriumi, Yamamoto, *et al*. reported that the crystallisation temperature of HfO_2_ was increased by the addition of lanthanum oxide (La_2_O_3_)^27,28^. The radius of the lanthanum ion is larger than that of the hafnium ion, and La_2_O_3_ serves to stabilize the amorphous structure in the HfO_2_–La_2_O_3_ film by disrupting the long-range order of crystalline HfO_2_. By the same logic, the crystallisation of Ta_2_O_5_ can also be suppressed by adding another element with a larger ionic radius. Fujikawa and Taga first reported an increase in the crystallisation temperature of Ta_2_O_5_ caused by addition of yttrium oxide (Y_2_O_3_)^29^; the effect on current leakage was also reported. However, the optical constants, such as *n* and the extinction coefficient (*κ*) of this film—a so-called amorphous tantalum yttrium oxide (aTYO)—were not reported. In other studies, an aTYO was used as a phosphor, and photoluminescence spectra were reported^29–33^, but there were no reports on the values of *n* and *κ* for the aTYO.

In this work, we prepared an aTYO using magnetron sputtering and investigated its electronic structure, crystallisation temperature, *n*, and *κ*. We also used the aTYO to fabricate a microcavity and compared the Faraday rotation and transmissivity of the fabricated microcavity with those of a previously fabricated microcavity using Ta_2_O_5_. We show that aTYO is a robust amorphous material suitable for integrated optical devices.

## Results

### Characterisation of Valence States

**Figure 1:**
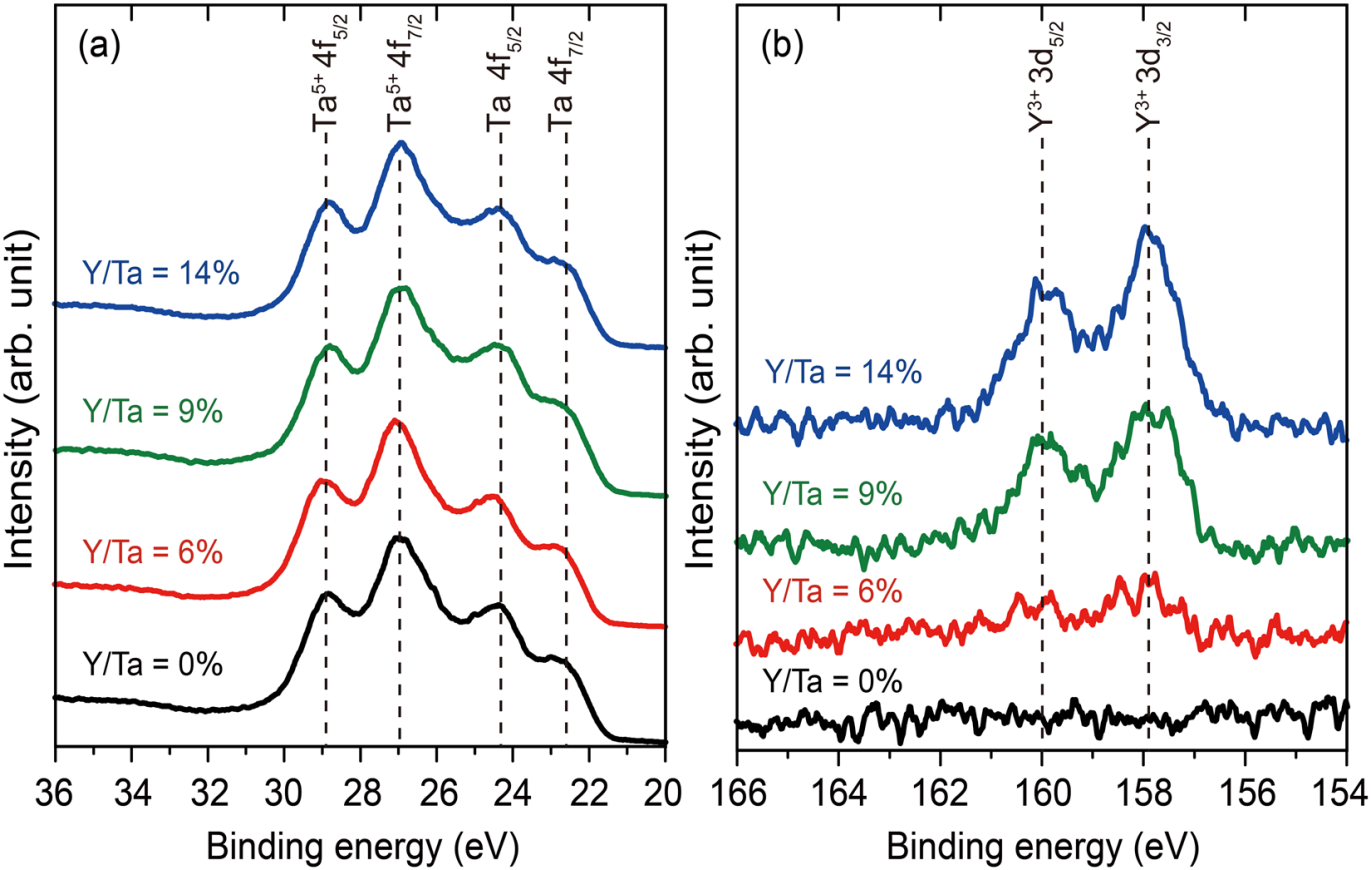
Valence states of the samples. XPS of the as-deposited aTYO and Ta_2_O_5_ films in the vicinity of the (**a**) Ta^5+^ 4 f, Ta 4 f and (**b**) Y^3+^ 3d energy levels. The film thickness was 180 nm, and the top 2 nm of each film was etched by Ar^+^ to remove the effects of changes in the oxidation state at the surface.

The X-ray photoelectron spectra (XPS; SXM-CI, ULVAC-PHI, Japan) of the as-deposited aTYO films and a Ta_2_O_5_ film were measured. Before the XPS measurements, the top 2 nm was etched from each film by Ar^+^ milling (bombardment) to remove the effects of surface stoichiometry changes on the oxidation state; the XPS peak positions were then calibrated so that the C 1s peak appeared at the binding energy of 284.8 eV^34^. Figure 1 shows the XPS spectra in the vicinity of the Ta^5+^ 4f, Ta 4f, and Y^3+^ 3d energy levels. In Fig. 1a, the Ta 4f peaks in the spectra of all samples appear at the same position as that for Ta_2_O_5_, where Y/Ta = 0%. The Ta peak was deconvoluted into four peaks, with contributions coming from the Ta^5+^ at 28.8 eV (4f_5/2_), 26.9 eV (4f_7/2_), and from metallic Ta at 24.3 eV (4f_5/2_) and 22.6 eV (4f_7/2_)^32,35^. The peaks formed a doublet owing to spin-orbit splitting^36^. The metallic states were attributed to reduction induced by Ar^+^ bombardment^37^. In Fig. 1b, the two peaks corresponding to Y 3d_5/2_ and Y 3d_3/2_ appear at the same positions (157.9 eV and 160.0 eV, respectively)^34^ regardless of the amount of yttrium in the aTYO. The peak positions of Y^3+^ 3d_5/2_ and Y^3+^ 3d_3/2_ for aTYO were previously reported to be 157.5 eV and 159.6 eV^32,38^, respectively, which are slightly lower energy than the peak positions obtained in this study; however, these results are consistent with the Y^3+^ found in Y_2_O_3_^34,39^ or YTaO_4_^32^. Changes in the Y/Ta atomic ratio from 6% to 14% did not affect the valence states of Y and Ta in aTYO.

### X-ray Characterisation

**Figure 2:**
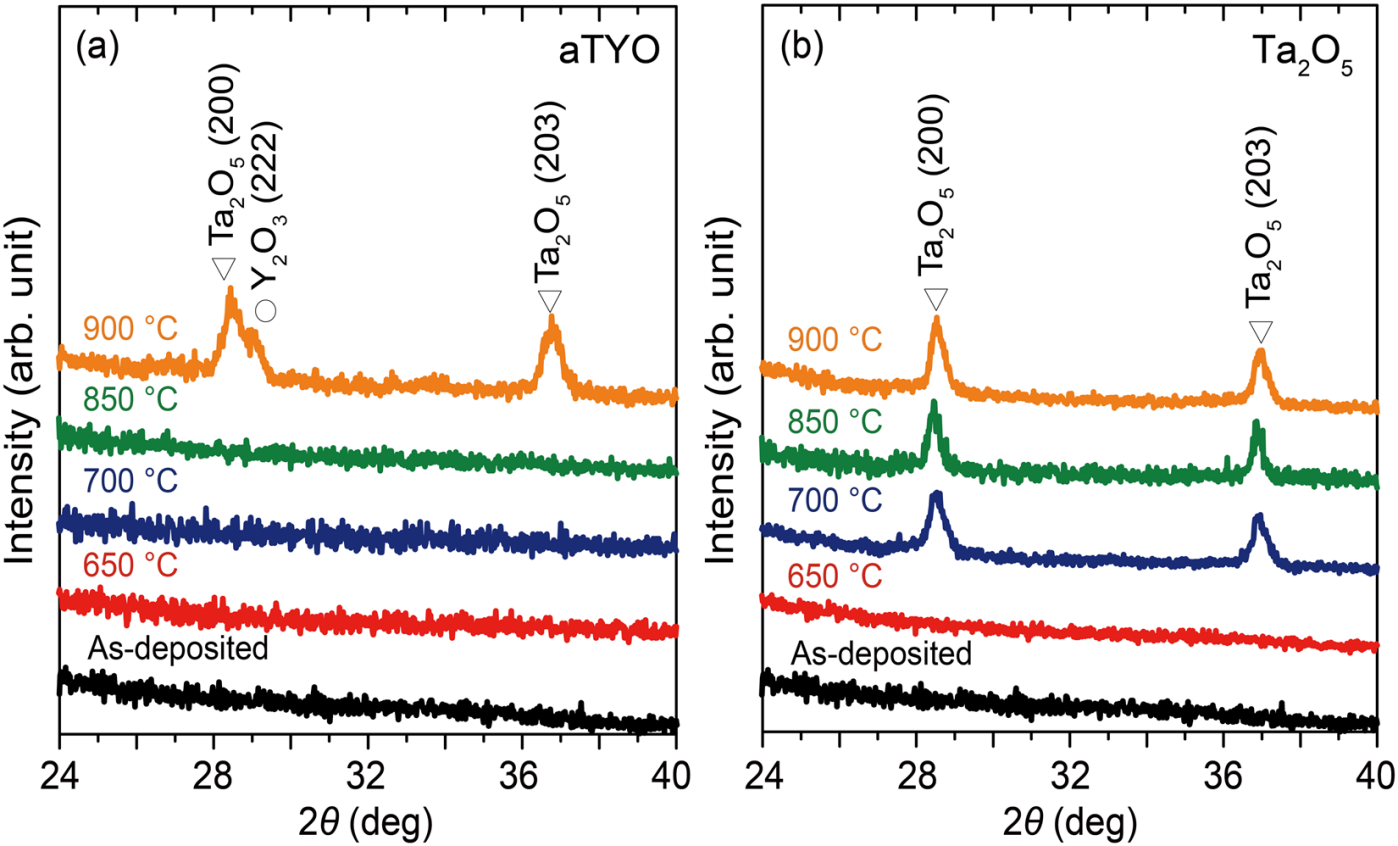
Crystalline states of the samples. XRD patterns of the annealed (**a**) aTYO films at 14% Y and (**b**) Ta_2_O_5_ films. Annealing was performed for 30 min in residual air at a pressure of 15 Pa (111 mTorr) and temperatures of 650 °C, 700 °C, 850 °C, or 900 °C. The white triangles indicate the diffraction peaks of Ta_2_O_5_^47^, and the white circle indicates the diffraction peak of Y_2_O_3_^54^.

The X-ray diffraction (XRD; RINT-2500, Rigaku, Japan) patterns of a Ta_2_O_5_ film and an aTYO films whose atomic fraction was 14% Y were measured to investigate their thermal stability after the addition of Y_2_O_3_ to Ta_2_O_5_. The films were annealed in a glass chamber at various temperatures for 30 min in residual air at a pressure of 15 Pa (111 mTorr), which was chosen because this is a suitable condition for the crystallisation of CeYIG on the BM^19^. A Cu Kα source at a wavelength of 0.1541 nm was used in the *ω*–2*θ* thin-film geometry XRD measurements, and the samples were tilted by 5° to eliminate the substrate peaks. Figure 2 shows the XRD patterns of aTYO and Ta_2_O_5_. The aTYO annealed at 900 °C exhibits peaks corresponding to those of Ta_2_O_5_ and Y_2_O_3_, but aTYO annealed at temperatures below 850 °C was in the amorphous state. The samples are too small to measure melting point and glass transition temperatures using thermogravimetric analysis or other bulk techniques, but they are described here as amorphous because they showed no nanocrystallinity by transmission electron microscopy (see below) and in XRD they exhibited a broad halo at 2*θ* ≈ 21° with a full width at half maximum from 9° to 17°, similar to as-grown Ta_2_O_5_ films. The observed halo position did not match any crystalline peaks.

The optimum annealing temperature of CeYIG on BM was 800 °C, which is within the range of stability for aTYO. However, Ta_2_O_5_ crystallised at temperatures above 700 °C. These results indicate that the crystallisation temperature of Ta_2_O_5_ increased by approximately 200 °C when Y_2_O_3_ was added to Ta_2_O_5_ at an atomic fraction of 14%, and the aTYO is sufficiently stable for integration in devices with CeYIG.

### Characterisation of Optical Constants

The transmissivity of the as-deposited and annealed aTYO films with 14% Y and the annealed Ta_2_O_5_ films was measured with a spectrometer (UV-3100PC, Shimadzu, Japan) to analyse the values of *n* and *κ* for each sample. The thicknesses of the as-deposited aTYO film, annealed aTYO film, and annealed Ta_2_O_5_ film were 320 nm, 320 nm, and 1060 nm, respectively. The annealing conditions were 30 min at 15 Pa residual air^40^. The spectra were fitted with SCOUT software ver. 3 (Techno Synergy, Japan), which calculated the optical interference based on the Fresnel equations.

**Figure 3:**
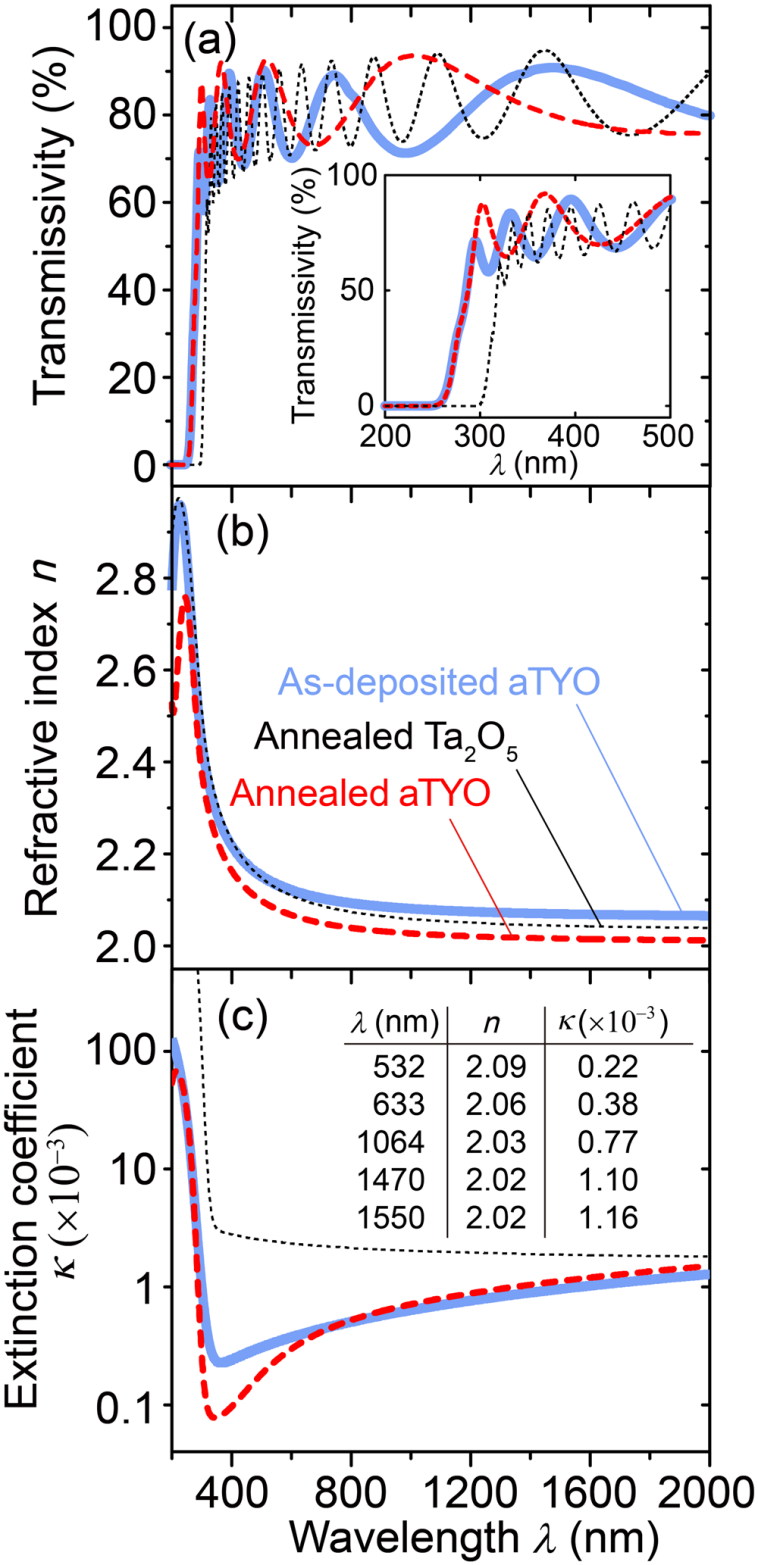
Optical properties of the samples. Plots of the (**a**) transmissivity, (**b**) refractive index (*n*), and (**c**) extinction coefficient (*κ*) as functions of the wavelength (*λ*), obtained by fitting the transmission spectra of each sample. Inset figure in (**a**) shows the enlarged short wavelength region. Inset table in (**c**) shows the *n* and *κ* of aTYO at representative wavelengths. Annealing was performed at 800 °C for 30 min in residual air at a pressure of 15 Pa.

Figure 3 shows plots of transmissivity, *n*, and *κ* as functions of wavelength. The addition of Y_2_O_3_ to Ta_2_O_5_ decreased *n*. At a wavelength of 1470 nm, the refractive indices of Y_2_O_3_, *n*_Y2O3_, annealed Ta_2_O_5_, *n*_Ta2O5_, and annealed aTYO, *n*_aTYO_, were 1.90^41^, 2.04, and 2.02, respectively. The *n*_aTYO_ value is close to the *n* = 2.02 calculated from the volume ratio of Y and Ta [=0.14 × *n*_Y2O3_ + 0.86 × *n*_Ta2O5_]. After annealing, the *n* of aTYO decreased. This might be because of expansion of the continuous random network^40,42^ of aTYO, as seen in other amorphous materials where the refractive index becomes smaller than the initial state after cooling down (quenching)^43–45^.

The *κ* value of annealed aTYO was also lower than that of annealed Ta_2_O_5_. At a wavelength of 1470 nm, the extinction coefficients of Y_2_O_3_, *κ*_Y2O3_, annealed Ta_2_O_5_, *κ*_Ta2O5_, and annealed aTYO, *κ*_aTYO_, were 7.22 × 10^−6^ ^41^, 1.89 × 10^−3^, and 1.10 × 10^−3^, respectively. The suppression of crystallisation reduced grain boundary contributions to loss, reducing the *κ* value^46^. The shift of the absorption edge of transmission is related to the electronic structure, specifically, the *d* orbitals of the transition metals^43^. The change of the Y^3+^ 3d_5/2_ state of aTYO, shown in Fig. 1b, might increase the band gap of the amorphous oxide.

These results indicate that the aTYO film is more stable against crystallisation than Ta_2_O_5_ while maintaining a high *n* and low *κ*. Although Y_2_O_3_ has a much lower *κ*, its low *n* means that a Bragg mirror made with Y_2_O_3_ would require more layers than one made with aTYO.

### Microcavity Structural Analysis

**Figure 4:**
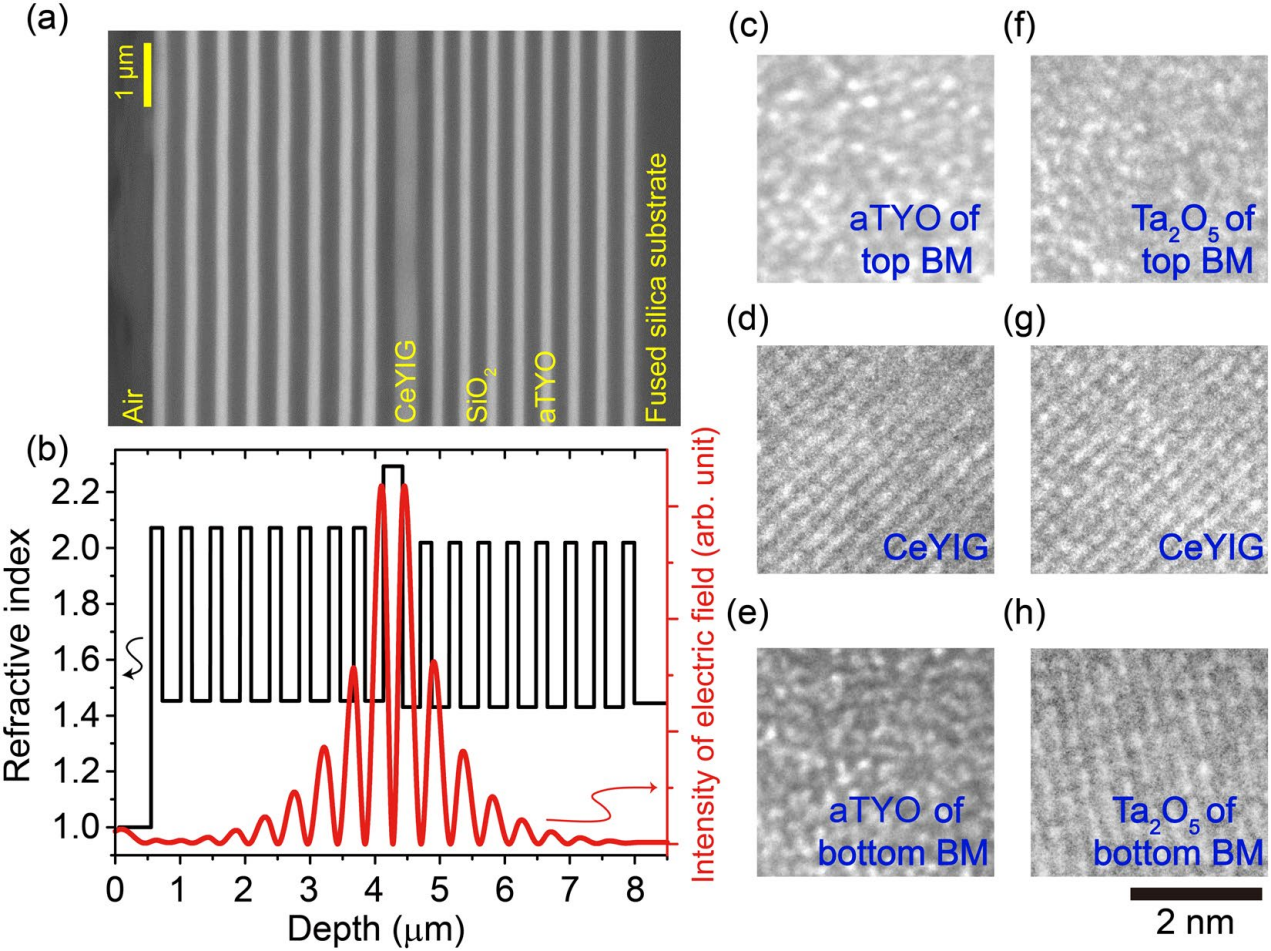
Microcavity structure. (**a**) Cross-sectional compositional image of a fabricated microcavity consisting of silica substrate/[(aTYO/SiO_2_)^8^]/CeYIG/[(SiO_2_/aTYO)^8^]. (**b**) Refractive index profile through the microcavity and intensity of the electric field calculated by the matrix approach. TEM images of (**c**) aTYO used in the top BM, (**d**) CeYIG used in the microcavity composed of aTYO, and (**e**) aTYO used in the bottom BM. TEM images of (**f**) Ta_2_O_5_ used in the top BM, (**g**) CeYIG used in the microcavity composed of Ta_2_O_5_, and (**h**) Ta_2_O_5_ used in the bottom BM.

Figure 4a shows a cross-sectional compositional image of the fabricated microcavity obtained with a field-emission scanning electron microscope (FE-SEM; JSM-6700F, JEOL, Japan) using back-scattered electrons, showing a clear layered structure. Not shown in this image, the film exhibited cracks with macroscale (10–100 μm) spacing. Figure 4b shows the profile of propagating light at a wavelength of 1470 nm, calculated as the squared intensity of the electric field in the microcavity using the matrix approach^20^. In this calculation, the actual thicknesses measured from Fig. 4a were used. The incident light was localized at the CeYIG defect layer, which breaks the periodicity of the BM. An enhancement of nonreciprocal effects was expected from this result because the localization increased the interaction between the light and the defect layer.

A transmission electron microscope (TEM; JEM-2100F, JEOL, Japan) was used to obtain images of the fabricated microcavity, in which aTYO or Ta_2_O_5_ was used in the BM. For the microcavity with aTYO-based BMs, Fig. 4c,d, and e show TEM images of the aTYO film used in the top BM, the CeYIG sandwiched between the two BMs using aTYO, and the aTYO film used in the bottom BM, respectively. For the microcavity with Ta_2_O_5_-based BMs, Fig. 4f,g, and h show TEM images of the Ta_2_O_5_ film used in the top BM, the CeYIG sandwiched between the two BMs using Ta_2_O_5_, and the Ta_2_O_5_ film used in the bottom BM, respectively. Figure 4d and g show the lattice fringes in the magnetic layer of each microcavity, indicating that the CeYIG was crystallised by annealing; the lattice spacings obtained from these images were 0.274 nm with a standard deviation (*σ*) of 0.008 nm and 0.281 nm with *σ* = 0.005 nm, respectively. Both numbers are close to the lattice spacing of 0.277 nm for the (420) plane of garnet, as calculated from the inorganic crystal structure database (ICSD) POWD-12++. The (420) diffraction peak shows the highest intensity of polycrystalline garnets. In contrast, no lattice fringes can be seen in Fig. 4c and f, indicating that the as-deposited aTYO and Ta_2_O_5_ were in the amorphous state.

An obvious difference between the two microcavities was observed in the bottom BM. As shown in Fig. 4e, the aTYO layers were amorphous. However, Fig. 4h shows fringes, indicating that the Ta_2_O_5_ layers were crystallised. The lattice spacing of Ta_2_O_5_ was 0.313 nm (*σ* = 0.003 nm), corresponding to the (200) plane of Ta_2_O_5_^47^, which has the highest peak intensity for obtained polycrystalline Ta_2_O_5_. Crystallisation is believed to cause scattering of light, degrading the transmissivity and lowering the *Q*-factor of the microcavity as discussed below.

**Figure 5:**
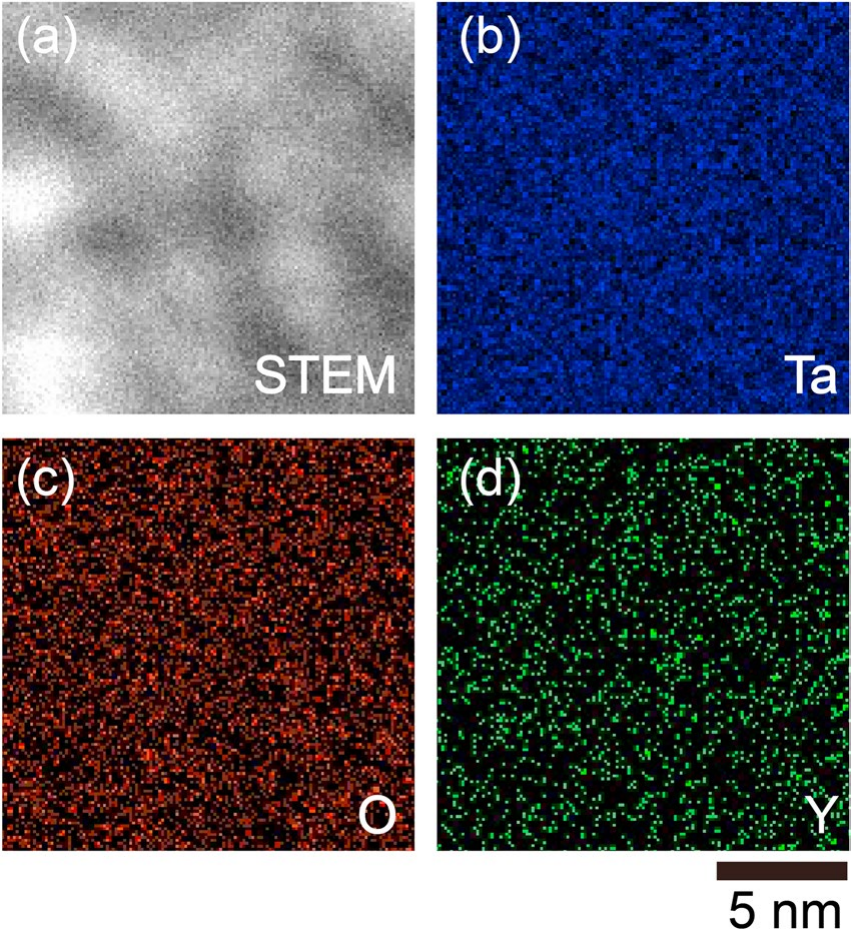
Compositional analysis. (**a**) STEM image of aTYO film used in the top BM. EDX mapping of (**b**) Ta, (**c**) O, and (**d**) Y.

We also carried out elemental mapping of the aTYO film by scanning transmission electron microscope and EDX (STEM–EDX) to investigate the distribution of elements. Figure 5a shows a STEM image of an aTYO film used in the top BM, and Fig. 5b,c, and d show EDX mappings of Ta, O, and Y, respectively. These figures indicate that Ta and O were uniformly distributed in the aTYO film, and Y was present in the aTYO. Figure 5a shows varying contrast, but this might be due to the roughness of the prepared sample. No evidence of nanocrystalline regions^48^, e.g., inhomogeneous distributions of specific elements, was observed, which is consistent with the XRD results.

### Magnetooptical Properties

**Figure 6:**
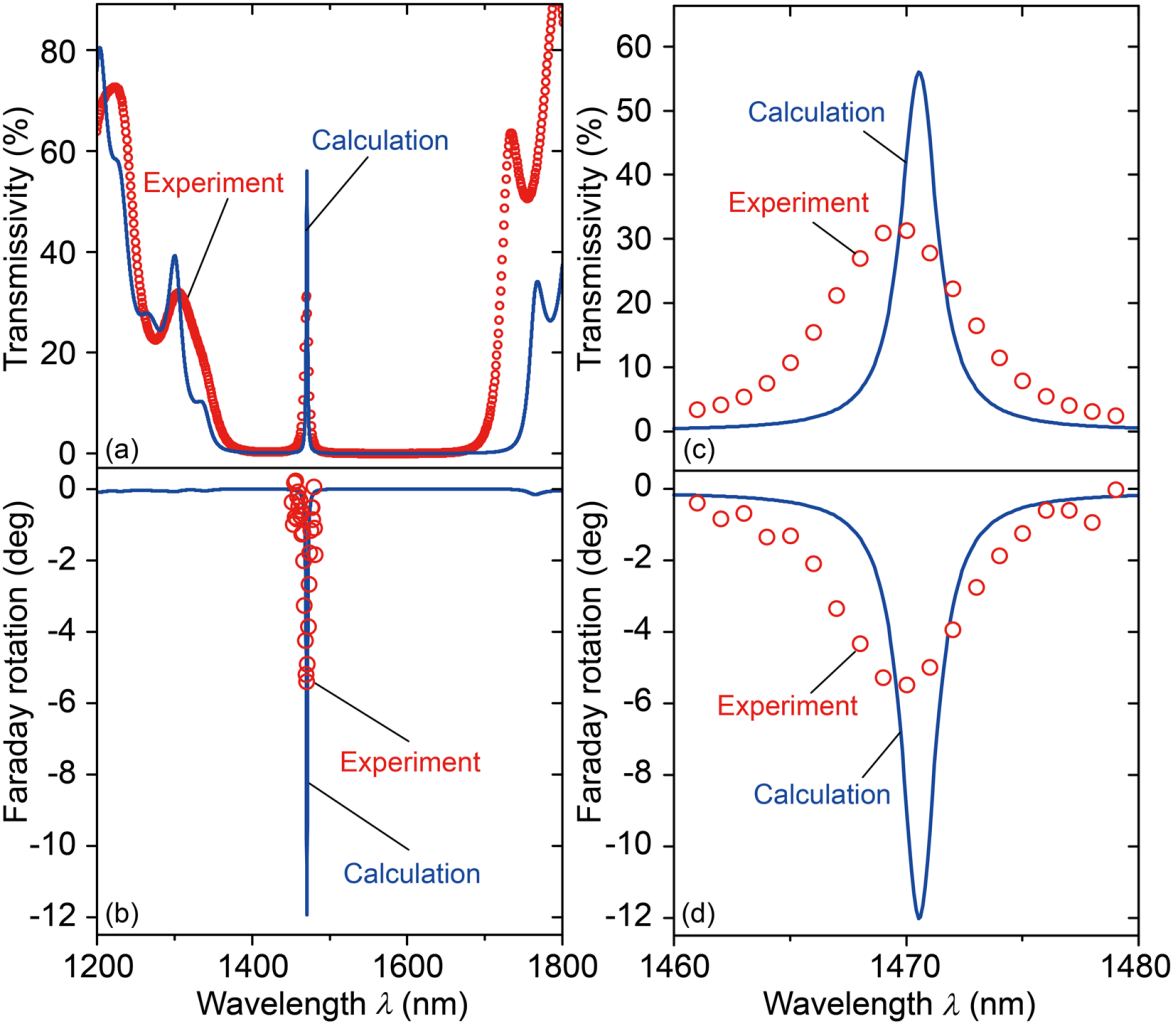
Optical and magnetooptical properties of the microcavity. (**a**) Transmission and (**b**) Faraday rotation spectra of the fabricated microcavity. (**c**,**d**) Enlarged figures of (**a**) and (**b**) in the vicinity of the peaks. The error bars are smaller than the symbol size.

Figure 6a shows the measured and calculated transmission spectra of the fabricated microcavities. The transmissivity was measured with a spectrometer and the Faraday rotation was measured with a rotating analyser (BH-M600VIR-FKR-TU, Neoark, Japan) by applying a magnetic field of 2 kOe (=160 kA/m) perpendicularly to the films^49^. The incident light at the surface of the sample had a spot size of approximately 2 mm. The halogen lamp was monochromated to give a spectral resolution of approximately 3 nm (the measured wavelength step was 1 nm). The localized mode was observed experimentally at a wavelength of 1470 nm, showing good agreement with the theoretical spectra calculated by the matrix approach^20^. At this wavelength, the propagating light has the profile shown in Fig. 4b; thus, localization in the iron garnet layer was confirmed. In these calculations, we used the actual thicknesses of each layer as determined by the cross-sectional SEM image shown in Fig. 4a and the optical parameters shown in Fig. 3. Other optical parameters of CeYIG, including *n* and *κ*, were taken from our previous report^19^.

Figure 6b shows an enhanced Faraday rotation of −5.4° and transmissivity of 31% at 1470 nm for the microcavity with aTYO-based BMs. Figure 6c and d show the response in more detail. Compared to the microcavity using Ta_2_O_5_ in the bottom BM, Faraday rotation was 1.8 times larger (−2.92°) and transmissivity was 10 times higher (3%) at the resonant wavelength of each microcavity^20^. However, the Faraday rotation and transmissivity of the aTYO-based microcavity were still low when compared to the calculation results. This is attributed to the variability of the thickness, *n*, and *κ* in the layers, to interface roughness, and/or to degradation in transmissivity caused by scattering from the cracks due to thermal mismatch. The broadening of the experimental peaks is attributed to a spatial low coherence within the light beam. Cracks and delamination of the CeYIG and upper layers were introduced after the annealing process. The estimated thermal mismatch strain at 800 °C for the CeYIG, Ta_2_O_5_, and SiO_2_ films were 0.8%, 0.5%, and 0%, respectively, as calculated using the thermal expansion constants of YIG (1.0 × 10^−5^ K^−1^)^50^, Ta_2_O_5_ (6.7 × 10^−6^ K^−1^)^51^, SiO_2_ (5.1 × 10^−7^ K^−1^)^52^, and the fused silica substrate (same as that of SiO_2_). Thermal mismatch strain can be reduced by using other substrate materials that have thermal expansion coefficients closer to that of CeYIG or by patterning the substrate so that the cracks form at specific places away from the device area. Nevertheless, these results show the potential of aTYO as a high-*n* material for the fabrication of optical devices using BMs with high thermal structural stability.

## Discussion

The electronic structure, crystallisation temperature, refractive index, and extinction coefficient of aTYO films fabricated by magnetron sputtering were analysed. When the Y/Ta atomic ratio of aTYO was 14%, the crystallisation temperature of aTYO was approximately 200 °C higher than that of Ta_2_O_5_. The extinction coefficient of aTYO annealed at 800 °C was lower than that of annealed Ta_2_O_5_ because the aTYO remained amorphous. A microcavity comprising CeYIG and aTYO-based BMs was fabricated as a demonstrative application of aTYO. The Faraday rotation and transmissivity were −5.4° and 31%, respectively, at a wavelength of 1470 nm. These values were respectively 1.8 and 10 times larger than those of a microcavity using crystallised Ta_2_O_5_ in the bottom BM. These results demonstrate the applicability of aTYO as a high-n material with high thermal stability against crystallisation; they also show the feasibility of using aTYO in BMs to fabricate optical devices resistant to annealing.

## Methods

### Preparation of aTYO Samples

Films of aTYO were fabricated on synthetic fused silica (hereafter, silica) substrates by radio-frequency (RF) magnetron sputtering (HSR-551S, Shimadzu, Japan) with various atomic ratios of yttrium/tantalum (Y/Ta). The sputtering target consisted of a Ta_2_O_5_ disk with a diameter of 10 cm (4 in), on which Y_2_O_3_ pellets were placed, and the number of Y_2_O_3_ pellets was changed to vary the Y/Ta atomic ratio. During deposition, Ar gas and O_2_ gas were introduced into the chamber at 8.0 cm^3^ min^−1^ and 2.0 cm^3^ min^−1^, respectively. The temperature of the substrate was kept constant at 250 °C, and an RF power of 75 W was applied to the target. The fabricated aTYO film had a thickness of 180 nm. The atomic fractions of the aTYO films were measured by energy-dispersive X-ray (EDX; JSM-6700F, JEOL, Japan) spectroscopy to be 0% (pure Ta_2_O_5_), 6%, 9%, and 14% Y.

### Preparation of the Microcavity Using aTYO Films in BMs

Sputtered aTYO films were used to fabricate a microcavity with the configuration of silica substrate/[(aTYO/SiO_2_)^8^]/CeYIG/[(SiO_2_/aTYO)^8^]. First, the bottom BM, comprising eight layers of aTYO/SiO_2_ (represented by [silica substrate/(aTYO/SiO_2_)^8^]), was prepared by ion-beam sputtering (IBS; OSI-WAVE-IBS, RMtec, Japan) because its deposition rate was higher than that offered by magnetron sputtering. The targets were an aTYO disk whose Y/Ta atomic ratio was 14% and a SiO_2_ disk, both of which had diameters of 10 cm (4 in). During the deposition, an RF power of 110 W was applied to the target, the substrate was held at 200 °C, and 7.5 cm^3^ min^−1^ of Ar gas and 6.0 cm^3^ min^−1^ of O_2_ gas were introduced into the chamber. The as-deposited aTYO and SiO_2_ had thicknesses of 265 nm and 165 nm, respectively. The center wavelength of the photonic band gap of the bottom BM was 1423 nm according to measurement with a spectrometer.

After the fabrication of the first BM, a 309 nm–thick polycrystalline CeYIG layer was prepared by magnetron sputtering. The film was deposited by applying an RF power of 75 W to a target with a diameter of 10 cm (4 in) in 1.3 Pa (10 mTorr) of Ar gas. The nominal composition of the target was Ce_1.0_Y_2.5_Fe_5.0_O_12–δ_ (δ shows oxygen deficiency). The substrate was held at 25 °C by water cooling during deposition. The as-deposited CeYIG film was amorphous, and the sample was annealed at 800 °C for 30 min in 15 Pa (111 mTorr) of residual air. After annealing, the top BM, comprising eight layers of aTYO/SiO_2_, was fabricated on the CeYIG by IBS using the deposition conditions of the bottom BM without further annealing. The aTYO and SiO_2_ used in the top BM had thicknesses of 276 nm and 188 nm, respectively. (The second SiO_2_ layer in the top BM was thinner, ~210 nm, because of a mistake during film preparation). The center wavelength of the photonic band gap of the top BM was 1574 nm. The differences in thicknesses and center wavelengths of the photonic band gap between the top and bottom BMs were attributed to several experimental factors. The deposition system was operated manually, leading to unintended variations in layer thickness; furthermore, the bottom BM was annealed, whereas the top BM was not. These issues could be ameliorated by using an *in-situ* thickness monitoring system^21^, or bonding of half of the microcavity^53^ to ensure identical top and bottom BMs.
